# High-intensity intermittent training ameliorates methotrexate-induced acute lung injury

**DOI:** 10.1186/s12890-024-02853-w

**Published:** 2024-01-20

**Authors:** Mohammad Amin Rajizadeh, Mahdiyeh Haj Hosseini, Mina Bahrami, Faegheh Bahri, Fahimeh Rostamabadi, Fatemeh Bagheri, Kayvan Khoramipour, Hamid Najafipour, Mohammad-Abbas Bejeshk

**Affiliations:** 1 Student Research Committee, Kerman University of Medical Sciences, Kerman, Iran; 2 Department of Physiology and Pharmacology, Kerman University of Medical Sciences, Kerman, Iran; 3 Physiology Research Center, Institute of Neuropharmacology, Kerman University of Medical Sciences, Kerman, Iran; 4 Department of Exercise Physiology, Faculty of Physical Education and Sports Sciences, Shahid Bahonar University of Kerman, Kerman, Iran; 5 Department of Clinical Biochemistry, Faculty of Medicine, Kerman University of Medical Sciences, Kerman, Iran; 6 Noncommunicable Diseases Research center, Bam university of medical sciences, Bam, Iran; 7 Department of Medical Immunology, Faculty of Medicine, Rafsanjan University of Medical Sciences, Rafsanjan, Iran; 8 Pathology and Stem Cell Research Center, Department of Pathology, Afzalipour School of Medicine, Kerman, Iran; 9 Cardiovascular Research Center, Institute of Basic and Clinical physiology Sciences, Kerman University of Medical Sciences, Kerman, Iran

**Keywords:** Methotrexate, High-intensity intermittent training, Acute lung injury, Oxidative stress, Inflammatory factors, Therapeutic effects, Mohammad Amin Rajizadeh, Mahdiyeh Haj Hosseini, Mina Bahrami, Faegheh Bahri and Fahimeh Rostamabadi equally contributed as first author.

## Abstract

Inflammation and oxidative stress are recognized as two primary causes of lung damage induced by methotrexate, a drug used in the treatment of cancer and immunological diseases. This drug triggers the generation of oxidants, leading to lung injury. Given the antioxidant and anti-inflammatory effects of high-intensity intermittent training (HIIT), our aim was to evaluate the therapeutic potential of HIIT in mitigating methotrexate-induced lung damage in rats. Seventy male Wistar rats were randomly divided into five groups: CTL (Control), HIIT (High-intensity intermittent training), ALI (Acute Lung Injury), HIIT+ALI (pretreated with HIIT), and ALI + HIIT (treated with HIIT).

HIIT sessions were conducted for 8 weeks. At the end of the study, assessments were made on malondialdehyde, total antioxidant capacity (TAC), superoxide dismutase (SOD), glutathione peroxidase (Gpx), myeloperoxidase (MPO), interleukin 10 (IL-10), tumor necrosis factor-alpha (TNF-α), gene expression of T-bet, GATA3, FOXP3, lung wet/dry weight ratio, pulmonary capillary permeability, apoptosis (Caspase-3), and histopathological indices.

Methotrexate administration resulted in increased levels of TNF-α, MPO, GATA3, caspase-3, and pulmonary edema indices, while reducing the levels of TAC, SOD, Gpx, IL-10, T-bet, and FOXP3. Pretreatment and treatment with HIIT reduced the levels of oxidant and inflammatory factors, pulmonary edema, and other histopathological indicators. Concurrently, HIIT increased the levels of antioxidant and anti-inflammatory factors.

## Introduction

Methotrexate (Mtx) is an anti-proliferative antagonist of folic acid used in the treatment of various diseases [1]. Patients treated with Mtx often suffer from its side effects, including an unproductive cough, shortness of breath, and pulmonary fibrosis [2]. Methotrexate-induced lung injury is a form of acute lung injury [3, 4]. Several pharmacological mechanisms have been proposed to explain the impact of Mtx, including the inhibition of purine synthesis, pro-inflammatory cytokine production, chemotaxis, neutrophil adhesion, and reduction of serum immunoglobulin. One of the hallmarks associated with the pathogenesis of Methotrexate-induced lung injury (MILI) is an increase in oxidative stress [5]. Additionally, acute lung damage results from inflammation [6].

Transcription factors such as T-bet and GATA3 play a role in the differentiation of Th1 and Th2 cells. The imbalance between Th1 and Th2 immune responses is one of the factors contributing to lung inflammation [7]. Mtx has been shown to decrease GSH and IL-10 levels while significantly increasing MPO, MDA, and TNF-α, which are indicators of inflammatory response [5]. Moreover, excessive use of Mtx can lead to the release of pro-inflammatory cytokines due to increased oxidative stress. It has been observed that many inflammatory diseases are associated with the dysfunction of regulatory T cells (Treg). The Foxp3 transcription factor is responsible for the differentiation and development of Treg [8, 9]. Based on these findings, it is anticipated that anti-inflammatory and antioxidant therapies would be beneficial in preventing Mtx-induced lung damage [10].

Short bouts of high-intensity exercise, alternating with rest intervals or moderate exercise, are known as high-intensity intermittent training (HIIT). This exercise regimen has been demonstrated to improve health-related quality of life [11, 12]. In contrast to low-intensity exercise, high-intensity exercise may be beneficial in eliciting a physiological exercise response. HIIT has been proposed as a more appealing approach than continuous exercise. With this intensity, the impact of lactic acid on arterial blood pH is far less than that with continuous exercise of moderate intensity [13]. Recent studies indicate that HIIT enhances pulmonary function and exercise tolerance in individuals with obstructive pulmonary diseases [14]. Therefore, this study aims to investigate whether HIIT can play a protective and/or therapeutic role in male rats with Mtx-induced lung damage.

## Materials and methods

### Animal groups and exercise training

The study involved 60 adults male Wistar rats (weight: 180–200 g; age: 8 weeks old) obtained from the Kerman University of Medical Sciences animal house. The study protocols were reviewed and approved by the University’s ethics committee (Ethics code: IR.KMU.REC.1400.367). All procedures were conducted following the ARRIVE guidelines for the care and use of laboratory animals. The rats were housed under standard conditions with 12 hours of light and a temperature maintained at 23 ± 2 °C. The rats were divided into five groups, each containing 12 rats: CTL Group: These rats did not undergo any exercise during the study and served as the control group. They were placed on the non-moving treadmill. HIIT Group: Intact rats underwent High-Intensity Intermittent Training (HIIT), consisting of 10 repetitions at a speed of 18 m per minute with a 30-degree slope, 5 days a week, for 8 weeks. Each repetition was followed by 1 minute of active rest (walking on a treadmill at a speed of 5 m per minute).

**Fig. 1:**
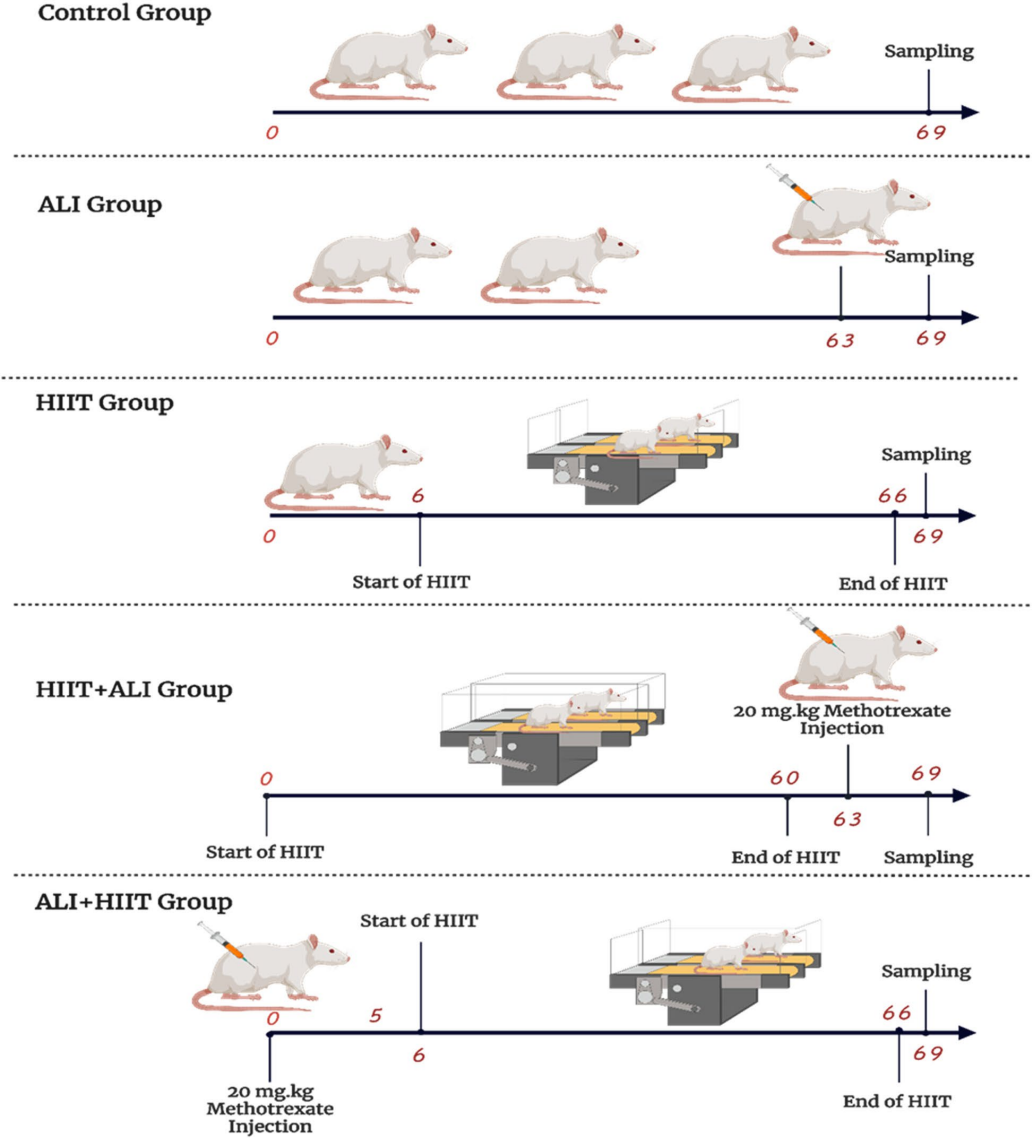
Timeline of animal Group and study design

ALI Group: Rats in this group received a single intraperitoneal injection of Mtx at a dose of 20 mg/kg to induce acute lung injury [4, 15]. HIIT+ALI Group: Rats underwent 8 weeks of HIIT followed by induction of acute lung injury using Mtx. ALI + HIIT Group: Rats were first subjected to acute lung injury by administering Mtx, followed by 8 weeks of HIIT [16] (see Fig. 1).

Five rats from each group were selected for the evaluation of pulmonary capillary permeability, while seven rats were chosen for other assays such as molecular and histological evaluations.

### Sample collection

In the ALI group, rats were sacrificed 5 days after Mtx administration. For the HIIT+ALI group, rats underwent 8 weeks of HIIT and then received Mtx. After 5 days, they were sacrificed. In the ALI + HIIT group, rats first received Mtx, and after 5 days, they underwent 8 weeks of HIIT. Animals in the HIIT and ALI + HIIT groups were sacrificed 48 hours after the last training session [4].

Following sacrifice (using a lethal dose of ketamine (80 mg/kg) and xylazine (50 mg/kg)), the chest was dissected, and lung tissue was extracted. The upper right lobe was immediately frozen in liquid nitrogen and stored at − 80 °C for molecular evaluation, while the lower right lobe was preserved in 10% formalin for subsequent histopathological and immunohistochemical assessments [17].

### Bronchoalveolar fluid (BALF) collection

After euthanizing the animals and removing the heart, the right lung was clamped, and 1 ml of phosphate-buffered saline (PBS) was injected using a syringe through a tracheal cannula into the left lung. Following a 2-minute interval, the infused liquid was lavaged and subsequently stored at − 80 °C [18].

### Evaluation of the lung edema

In a distinct subgroup of animals, the lower lobe of the left lung was excised and weighed promptly at the conclusion of the study. The tissue was incubated at 60 °C for 48 hours to determine the dry weight. Subsequently, the ratio of the wet weight to the dry weight was calculated [19].

### Assessment of pulmonary capillary permeability

To assess pulmonary capillary permeability, Evans blue dye was used. A dose of 20 mg/kg of 2% Evans blue (Sigma Co.) was injected into the jugular vein. After 15 minutes, the animals were sacrificed, and their lungs were excised and weighed. Subsequently, the lung tissue was incubated with 4 ml of formamide for 24 hours at 37 °C to extract the dye from the tissue. The intensity of color was measured using spectrophotometry at 620 nm, and a standard curve was utilized to determine the color value. The results were expressed as milligrams of dye per gram of tissue [20].

### Quantitative real-time PCR (qPCR)

The expression levels of T-bet, GATA3, and FOXP3 were assessed using SYBR Green real-time PCR. Total RNA was extracted from the samples employing Trizol reagent (Karmania Pars Gene, Iran). Subsequently, cDNA was synthesized following the manufacturer’s instructions using the Karmania Pars Gene Kit (Iran) [21]. The primer sequences utilized in this study are detailed in Table 1.

**Table 1:** Sequence of primers used in Real Time PCR

Name	Sequence (5′-3′)	Length
T-bet F	TCTTCCCTCCCAGCAGCCTAC	20
T-bet R	CAGTACCATCTCGCCGCCAC	21
GATA3 F	AAGTTCAACCAGCACCAGAC	21
GATA3 R	TCCACCAAGACTACATCCACA	20
FOXP3 F	TTGCCATCAACGACCCCTTCA	20
FOXP3 R	AGCACCAGCATCACCCCATTT	21
GAPDH F	TTCACCTATGCCACCCTCAT	21
GAPDH R	ACTGCTCCCTTCTCACTCTCC	21

### Measurement of oxidative stress Indicator

The level of malondialdehyde (MDA) in homogenized lung tissue was determined based on the formation of thiobarbituric acid reactants. Additionally, the total antioxidant capacity (TAC) of the homogenized lung tissue was assessed using the ferric-reducing antioxidant power (FRAP) measurement method. Superoxide dismutase (SOD) activity was evaluated using the calorimetric method with a specific kit (Navand Salamat Co.). Furthermore, the assessment of glutathione peroxidase was conducted utilizing a dedicated kit (Navand Salamat Co.) [22, 23].

### Myeloperoxidase (MPO)

The lung tissue myeloperoxidase (MPO) level serves as an indicator of neutrophil accumulation in the lungs. The assessment of lung tissue MPO was conducted using a specific kit provided by Navand Salamat Co. [24].

### Measurement of inflammatory indicators

TNF-α and IL-10 levels were quantified in both tissue homogenate and bronchoalveolar lavage fluid (BALF) following the instructions provided by specific ELISA kits obtained from Karmania Pars Gene Co.

### Histopathology

The left lung obtained from each experimental group underwent perfusion with 10% formaldehyde. Following perfusion, the samples were dehydrated and subsequently embedded in paraffin. Sections with a thickness of 4 μm were prepared and subjected to hematoxylin and eosin (H&E) staining. An unbiased pathologist evaluated the extent of lung injury in the H&E stained sections using a 5-point scoring system: (0) No change, (1) Mild, (2) Moderate, (3) Severe, (4) Very severe lung injury. This assessment was performed without knowledge of the animal groups, ensuring objectivity. The grading of lung injury was based on the evaluation of histological features [25, 26].

### Immunohistochemistry (IHC)

The samples were initially fixed in formalin overnight and subsequently dehydrated using various ethanol concentrations. Following this, the samples were embedded in molten paraffin and cut into 5-μm thick sections at random. To remove the paraffin, the sections were placed in an oven at 74 °C for 15 minutes. For blocking nonspecific binding sites, the sections were incubated with a blocking solution at room temperature for 40 minutes. Tissue incubation with a primary monoclonal antibody against Caspase-3 antibody was carried out overnight at 4 °C. Afterward, the cells were washed with PBS buffer and then incubated for 1 hour at room temperature in a dark environment with the corresponding secondary antibody. Following this step, the samples were washed three times with PBS buffer and stained with Hoechst (5 g/ml) at room temperature for 15 minutes. Subsequently, the slides were imaged using light microscopy [4, 24].

### Statistical analysis

The statistical analysis was conducted using Graph Pad Prism v.6 software. Initially, the normal distribution of the data was assessed using the Shapiro-Wilk test. For normally distributed quantitative data, comparisons were made using one-way ANOVA followed by Tukey’s post hoc tests. A significance level of *p* < 0.05 was considered statistically significant for all analyses.

## Results

### Lung edema and lung capillary permeability

**Fig. 2:**
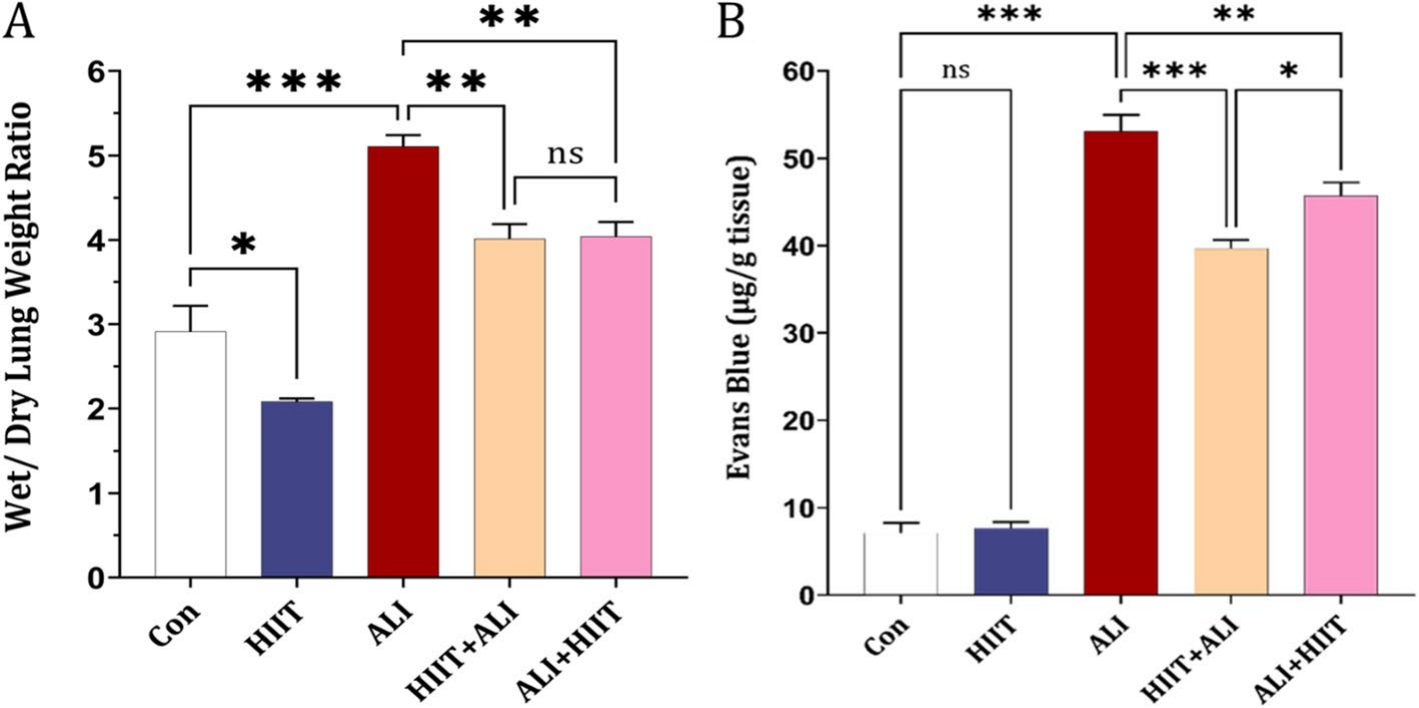
Effect of HIIT on (**a**) Wet/Dry lung weight ratio *n* = 7 in each group, (**b**); permeability of lung capillaries. Data are presented as mean ± SD for *n* = 5 in each group. * *p* < 0.05, * *p* < 0.01 and *** *p* < 0.001

Our findings indicated an increase in both the Wet/Dry lung weight ratio and Pulmonary capillary permeability (Evans Blue extravasation) in the ALI group when compared to the control group. However, pre-treatment and subsequent treatment with HIIT in the HIIT+ALI and ALI + HIIT groups led to a reduction in these indicators. Specifically, there was a more pronounced reduction in Pulmonary capillary permeability observed in the HIIT+ALI group in comparison to the ALI + HIIT group (refer to Fig. 2 A and B).

### Cytokines measurement

**Fig. 3:**
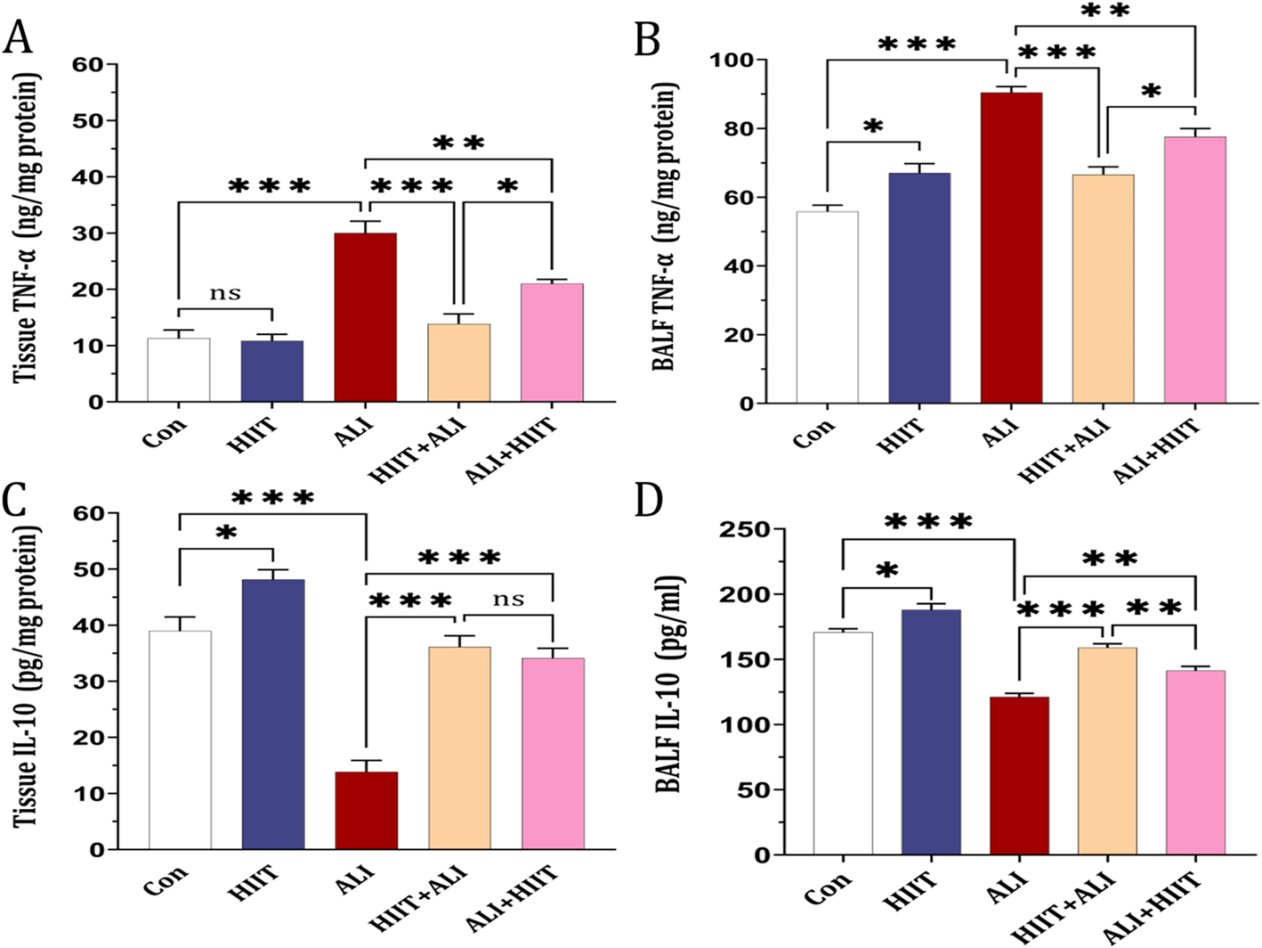
The Effect of HIIT on TNFα and IL-10 levels in lung tissue and BAL fluid. Data are presented as mean ± SD and *n* = 7 in each group. *P < 0.05 and ***P < 0.001 vs Con. ζζ *P* < 0.01 and ζζζ *P* < 0.001 vs ALI. ± *P* < 0.05 vs Post

The levels of Tissue and BALF TNF-α notably increased in the ALI group compared to the control group. However, a substantial reduction was observed in both the HIIT+ALI and ALI + HIIT groups compared to the ALI group (see Fig. 3 A and B). Notably, a significant difference was observed between the HIIT+ALI and ALI + HIIT groups regarding TNF-α levels.

Moreover, in the ALI group, both tissue and BALF IL-10 levels were markedly lower compared to the control group, but these levels were normalized in the HIIT+ALI and ALI + HIIT groups. A significant difference was also observed between the HIIT+ALI and ALI + HIIT groups in terms of BALF IL-10 levels (see Fig. 3 C and D).

### MPO in lung tissue

**Fig. 4:**
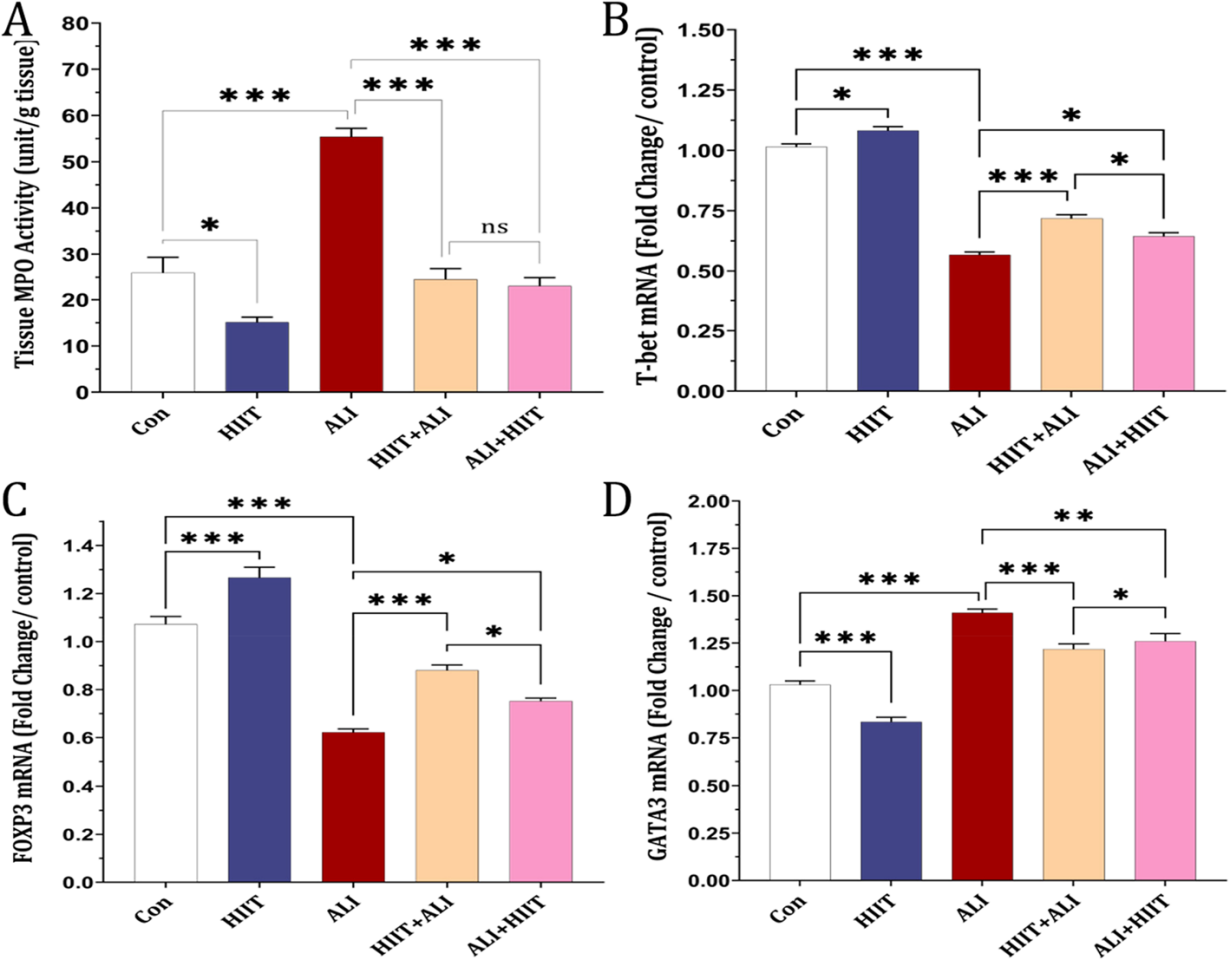
Effect of HIIT on (**a**), MPO, (**b**), Tbet mRNA expression, (**c**), FOXP3 mRNA expression, (**d**), GATA3 mRNA expression in lung tissue. Data are presented as mean ± SD for *n* = 7 in each group. * *p* < 0.05, * *p* < 0.01 and *** *p* < 0.001

The MPO level significantly increased in the ALI group compared to the control group. However, pre-treatment and subsequent treatment with HIIT in both the HIIT+ALI and ALI + HIIT groups led to a reduction in MPO levels (see Fig. 4A).

### T-bet, FOXP3 and GATA3 mRNA expression in lung tissue

Our findings demonstrated a reduction in mRNA expression of T-bet and FOXP3, along with an elevation in mRNA expression of GATA3 in the ALI group compared to the control group. However, pre-treatment and subsequent treatment with HIIT in both the HIIT+ALI and ALI + HIIT groups resulted in increased T-bet and FOXP3 mRNA expression and decreased GATA3 mRNA expression in comparison to the ALI group. Notably, the pretreatment phase showed more significant alterations than the treatment phase (see Fig. 4B-D).

### MDA, TAC, Gpx, SOD in BALF and lung tissue

**Fig. 5:**
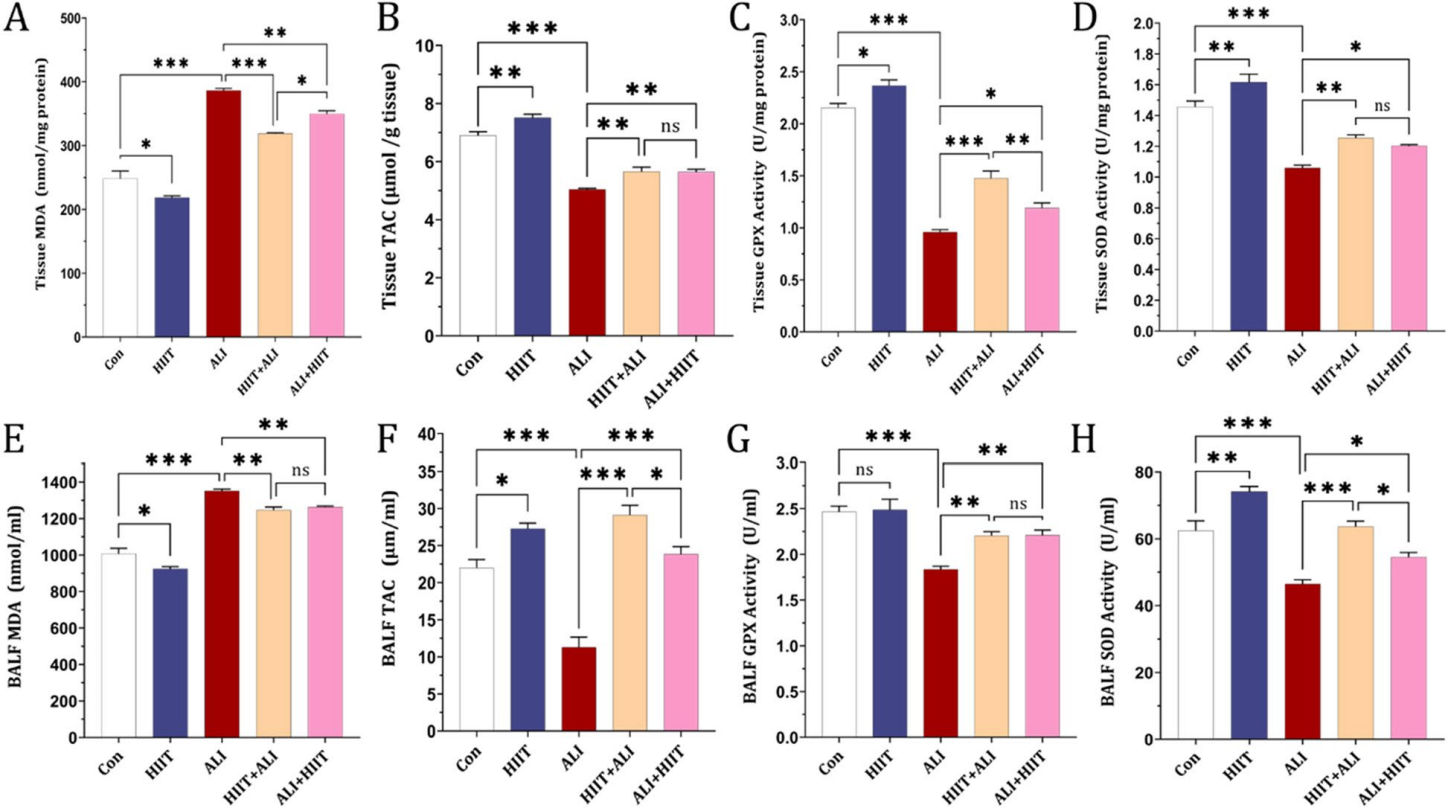
Effect of HIIT on (**a**); MDA, (**b**); TAC, (**c**); GPX, (**d**); SOD in lung tissue. Effect of CT on (**e**) MDA, (**f**); TAC, (**g**); Gpx, (**h**); SOD in BALF. Data are presented as mean ± SD for *n* = 7 in each group. * *p* < 0.05, * *p* < 0.01 and *** *p* < 0.001

In the ALI group, both tissue and BALF MDA levels were elevated compared to the control group. Notably, MDA levels decreased in both the HIIT+ALI and ALI + HIIT groups in comparison with the ALI group. Moreover, the tissue MDA level was lower in the HIIT+ALI group compared to the ALI + HIIT group (refer to Fig. 5 A and E).

Regarding lung tissue and BALF antioxidant factors, all three antioxidants TAC, GPX, and SOD showed remarkable reductions in levels in the ALI group compared to the control group. However, pre-treatment and subsequent treatment with HIIT resulted in an increase in the levels of all these antioxidants compared to the ALI group (see Fig. 5 B-D and F-H).

### Histopathological finding

**Fig. 6:**
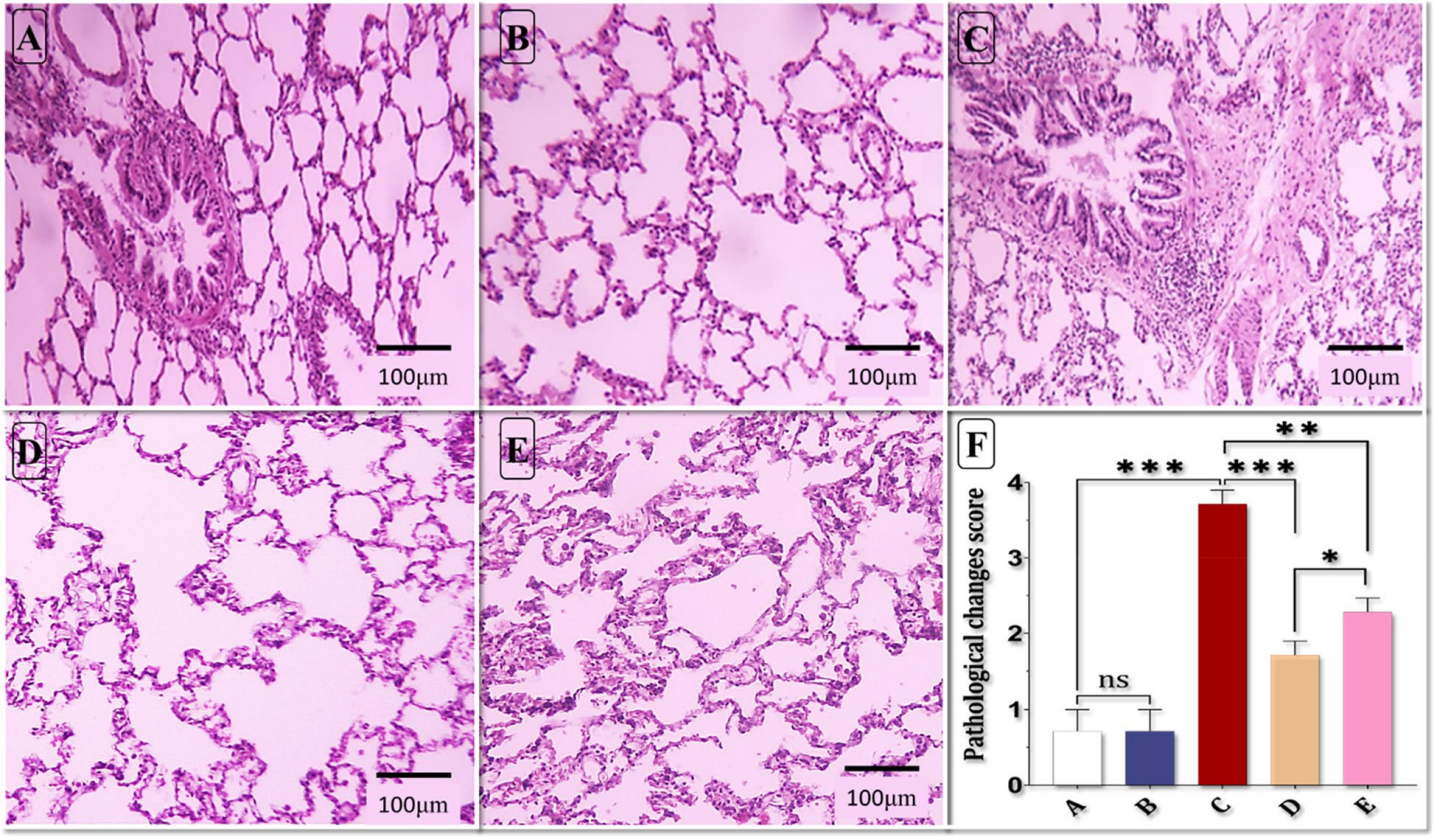
Effect of HIIT on microscopic presentation of Methotrexate-induced Acute Lung Injury in rats stained by hematoxylin and eosin (light microscopy, 10 X), **a** Control group, **b** High-intensity intermittent training group, **c** Acute Lung Injury group, **d** HIIT+ALI group, **e** ALI + HIIT group, **f** pathological changes score. Data are presented as mean ± SD for n = 7 in each group. * *p* < 0.05, * *p* < 0.01 and *** *p* < 0.001

The score reflecting lung pathological changes notably increased in the ALI group compared to the control group. However, both pre-treatment and subsequent treatment with HIIT significantly reduced the pathological changes induced by Mtx. Remarkably, the pretreatment phase exhibited more significant improvements compared to the treatment phase (see Fig. 6 A-F).

### Apoptosis of lung tissue

**Fig. 7:**
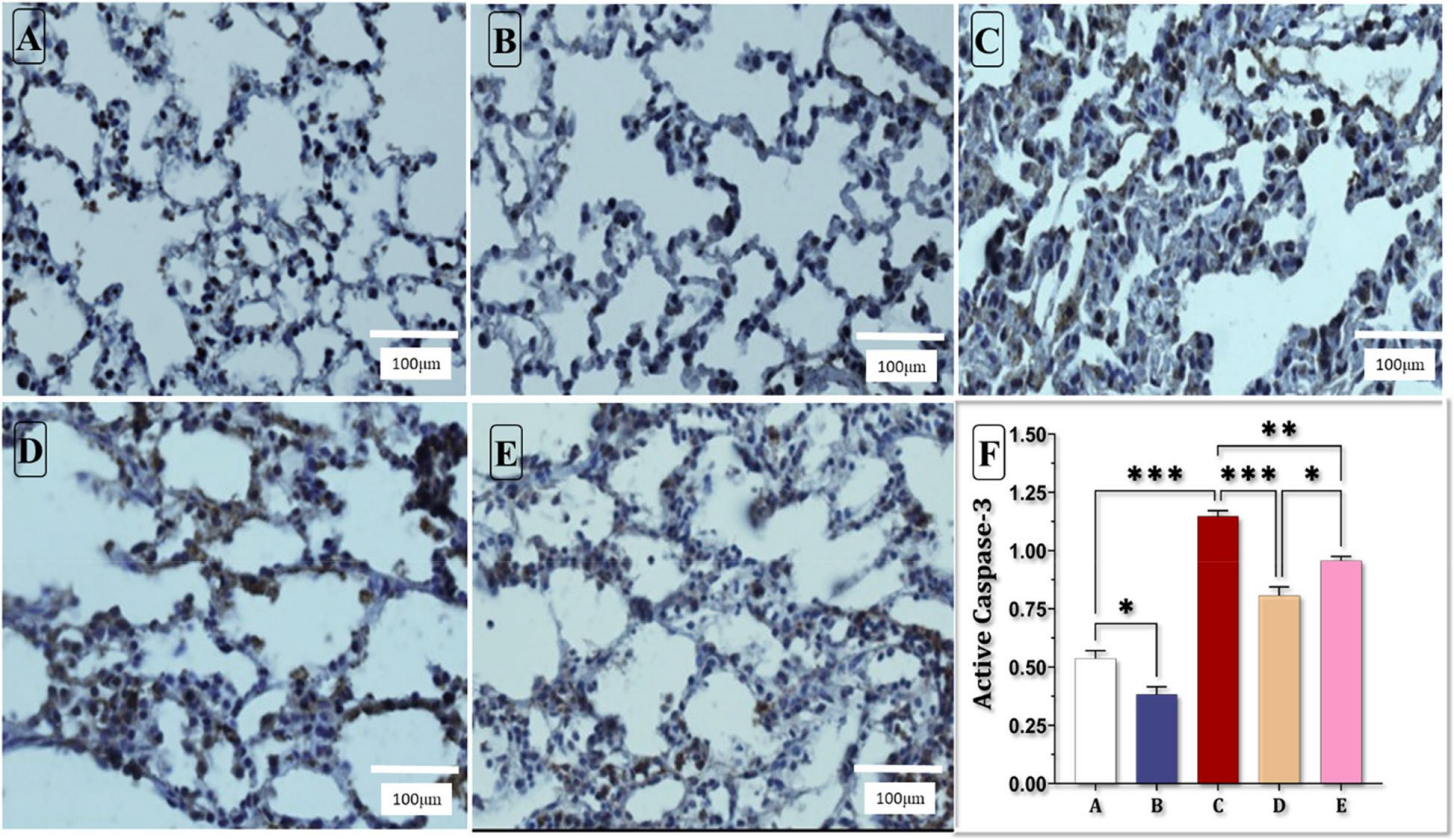
Immunohistochemical evaluation of the effect of HIIT on Caspase-3 expression (A-F) in lung tissue of rats. **a** Control group, **b** High-intensity intermittent training group, **c** Acute Lung Injury group, **d** HIIT+ALI group, **e** ALI + HIIT group, **f** Quantitative chart of expression of Active caspase-3 in lung tissue.* *p* < 0.05, ** *p* < 0.01, *** *p* < 0.001

Our immunohistochemical evaluation revealed a significant increase in active caspase-3 levels in the ALI group compared to the control group. However, both pre-treatment and subsequent treatment with HIIT in the HIIT+ALI and ALI + HIIT groups led to a decrease in active caspase-3 levels compared to the ALI group. Notably, the pretreatment phase exhibited more remarkable reductions than the treatment phase (see Fig. 7 A-F).

## Discussion

The objective of this investigation was to evaluate the impact of High-Intensity Interval Training (HIIT), both before and after injury, on inflammatory factors, oxidative stress, and pathological markers in methotrexate-induced lung injury in rats. The findings demonstrated that methotrexate (Mtx) elevated the levels of TNFα and IL-6 while reducing the levels of IL-10. However, in the HIIT-treated groups, the levels of TNFα and IL-6 were lower, and the level of IL-10 was higher in comparison to the control (non-treated) group.

In our prior research, we demonstrated the protective effects of continuous aerobic exercise against methotrexate-induced lung injury [25]. In this study, our focus was to evaluate the impact of High-Intensity Interval Training (HIIT), characterized by brief periods of intense anaerobic exercise alternated with short recovery periods, on methotrexate-induced lung injury. HIIT involves repeated bursts of high-intensity or explosive exercise followed by rest or low-activity intervals until the point of exhaustion. Its distinctive features, including very high intensity, interval duration, and repetitions, differentiate HIIT from traditional aerobic activities [27].

The constraint of time is a common obstacle preventing individuals from adhering to conventional training programs like continuous training. Consequently, sedentary lifestyles contribute significantly to metabolic-related disorders, posing a substantial health challenge in modern society. In this context, it is intriguing that brief yet highly intense exercise regimens may yield comparable enhancements in cardiorespiratory fitness and oxidative capacity as prolonged training sessions [28].

Houshmand Moghadam et al. demonstrated a significant reduction in serum inflammatory cytokines in the HIIT group compared to their control group [20]. However, unlike our study, they reported a notable increase in serum IL-8 levels [20]. The observed discrepancies might be attributed to variations in the study sample characteristics or the duration of the HIIT intervention between the two studies. Specifically, in Houshmand Moghadam et al.’s research, the HIIT period spanned 12 weeks, and their study participants consisted of overweight/obese individuals.

In our study, we observed a decrease in the expression levels of T-bet and Foxp3 genes, coupled with an increase in the GATA3 gene expression, in lung damage induced by methotrexate. These findings were consistent with Wei GU et al.’s research, which reported similar alterations in gene expression associated with lung injury [22]. However, in our study, the groups treated with HIIT showed increased levels of T-bet and Foxp3 transcription factors and decreased levels of GATA3 transcription factors.

Intense exercise has been associated with heightened anti-inflammatory factors. Studies by Yeh et al. and Wang et al. demonstrated an increase in Foxp3+ regulatory T cells following periods of exercise [23, 29]. Moreover, Hemmati et al.’s investigation on the impact of 12 weeks of exercise training on the immune system revealed exercise’s ability to inhibit inflammatory cytokines and its significant effects on immune modulation. Their findings indicated an elevation in T-bet gene expression and a decrease in the cytokine TNF-α; however, no change was observed in the expression of GATA-3, ROR γt, and FOXP3 genes, potentially attributed to the nature or type of exercise employed [30].

In our current research, an increase in MPO levels, indicating the infiltration of multinucleated leukocytes, was observed in methotrexate-induced lung injury. MPO accumulation is known to influence inflammatory pathways through the metabolism of nitric oxide (NO), arachidonic acid, and linoleic acid-derived mediators [31, 32]. Studies by Arpag et al. [31] and Mammadov et al. [33] linked high MPO levels to oxidative damage induced by methotrexate in the lungs.

Our findings indicated that HIIT reduced oxidative stress factors and lowered MDA levels in the HIIT group compared to the control group. Groussard et al. [34] also reported a reduction in oxidative agents in the HIIT group. Similarly, recent research by Nakao et al. [35] highlighted HIIT’s role in regulating antioxidant enzyme activity through NRF2 signaling. While some studies emphasized treadmill exercise preconditioning’s attenuation of lung damage following exposure to LPS [36–38], others suggested exercise after lung injury as a protective measure [39, 40].

However, contrasting findings were noted in studies by Groussard et al. [34], where SOD activity decreased in the rat liver following HIIT, and Rahmani et al. [41] reported increased serum levels of TAC and MDA in the HIIT group. These differences might stem from variations in the duration or intensity of HIIT between studies.

Our research uniquely demonstrates the beneficial effects of HIIT both before and after lung injury induced by methotrexate, shedding light on its potential in mitigating lung injury. It appears that exercise exerts protective effects by reducing inflammation and oxidative stress in this model of lung injury.

## Conclusion

The study results demonstrate that an eight-week regimen of high-intensity interval training effectively mitigated methotrexate-induced acute lung injury by rebalancing antioxidant/oxidant levels and modulating anti-inflammatory/inflammatory factors. Notably, the preventive effects of high-intensity exercise were more pronounced than its therapeutic effects in countering MTX-induced lung injury. This finding underscores the importance of adopting an active lifestyle as a preferable solution over solely relying on exercise as a treatment method for lung-related diseases.

### Limitations

An important limitation of our study was the lack of measurement of crucial cytokines such as IL17 or iNOS expression. Additionally, the absence of animal lung function tests represents another limitation in our work. Incorporating these measurements could have provided further valuable insights into the comprehensive understanding of the observed effects.

## Data Availability

The datasets used and/or analyzed during the current study are available from the corresponding author on reasonable request.
